# A Method for Reviewing the Accuracy and Reliability of a Five-Level Triage Process (Canadian Triage and Acuity Scale) in a Community Emergency Department Setting: Building the Crowding Measurement Infrastructure

**DOI:** 10.1155/2012/636045

**Published:** 2012-01-11

**Authors:** Michael K. Howlett, Paul R. T. Atkinson

**Affiliations:** ^1^Department of Emergency Medicine, Dalhousie University, Halifax, NS, Canada B3H 3A7; ^2^Saint John Regional Hospital, Saint John, NB, Canada E2L 4L2

## Abstract

*Objectives.* Triage data are widely used to evaluate patient flow, disease severity, and emergency department (ED) workload, factors used in ED crowding evaluation and management. We defined an indicator-based methodology that can be easily used to review the accuracy of Canadian Triage and Acuity Scale (CTAS) performance. *Methods.* A trained nurse reviewer (NR) retrospectively triaged two separate month's ED charts relative to a set of clinical indicators based on CTAS Chief Complaints. Interobserver reliability and accuracy were compared using Kappa and comparative statistics. *Results.* There were 2838 patients in Trial 1 and 3091 in Trial 2. The rate of inconsistent triage was 14% and 16% (Kappa 0.596 and 0.604). Clinical Indicators “pain scale, chest pain, musculoskeletal injury, respiratory illness, and headache” captured 68% and 62% of visits. *Conclusions.* We have demonstrated a system to measure the levels of process accuracy and reliability for triage over time. We identified five key clinical indicators which captured over 60% of visits. A simple method for quality review uses a small set of indicators, capturing a majority of cases. Performance consistency and data collection using indicators may be important areas to direct training efforts.

## 1. Introduction

Accurate assessment of triage (a French term meaning “to sort”) processes and outcomes is central to any research on emergency department (ED) crowding. Patient volumes and acuity, the aging population, public demands for advanced technology, evidence-based medicine, and payor austerity measures increase pressure on emergency department processes. With increasing demand on EDs and increasing crowding issues, studies must examine factors that influence patient flow. These studies depend on accurate measurements of disease severity and workload. Triage data are used extensively as a proxy for both workload and patient acuity. Thus proper conduct of research into crowding and the ability to apply results across facilities depends on accuracy and reliability of the data.

The five-level Canadian Triage and Acuity Scale (CTAS), using a standardized data element set including a validated Chief Complaint list, has been adopted by the Canadian Association of Emergency Physicians (CAEPs) and National Emergency Nurses Affiliation (NENA) as the standard methodology for emergency department triage [1–3]. Similar five-level systems are used in the United States, the United Kingdom, and Australia as well as in other jurisdictions. The Canadian Triage and Acuity Scale (CTAS) has been used not only for determining the priority by which patients are evaluated by physicians but also as a proxy for disease severity, nurse and physician workload, a tool in quality improvement processes, and in funding methodologies. Evaluation of the accuracy and reliability of CTAS performance becomes essential for reliable information.

CTAS performance has been measured through representative case evaluation and scoring by triage nurses, indirect measurement of ED statistics comparability between centers, prospective independent scoring in a live environment by duplicated triage staff, and by interobserver reliability studies. CTAS can be performed with very good interobserver reliability and moderately predicts the need for urgent care and resources [4–7]. Most studies to validate triage scoring are sophisticated double triage prospective designs, are difficult to apply across systems in real life, contain an intrinsic bias toward better performance, use classroom-based testing methods, do not address one or the other of accuracy or reliability, or do not pave the way for outcome-based decisions. In this study we evaluate an inexpensive and easy method to assess CTAS performance in a community emergency department (ED) using (1) a limited set of clinical indicators to allow manageable data analysis, and (2) a nurse reviewer trained in the CTAS National Guidelines (CAEP/NENA). The goal is more reliable performance of the triage function with more predictable effects on outcomes. This in turn would improve the ability to capture variations in systems and better evaluate how crowding influences outcome on a macrosystemic scale. This information ultimately may lead to interventions that improve care and reduce crowding.

## 2. Methods

The study was approved by the local Research Ethics Committee. Triage in this hospital was routinely performed by an experienced group of ED nurses who had been given CTAS training in the past and had national CTAS Guidelines materials including the CEDIS Chief Complaint list, a validated set of chief complaints for ED triage [8]. An experienced emergency nurse underwent further specific training with CAEP/NENA training materials to become the study's nurse reviewer (NR). The NR retrospectively triaged two separate month's ED charts one year apart blinded to physician data and triage score. The second data collection was performed one year later to determine the reproducibility of results after staff turnover occurred (approximately 25% of triage staff). Turnover led to the requirement that all triage staff undergo Continuing Education on the CTAS guidelines during the year between the two trials (one-day seminar using the same CAEP/NENA materials and the CEDIS Chief Complaint List).

Inter-observer reliability (IR) was performed between the original charted CTAS score and the NR score. IR results were compared using raw and quadratic weighted Kappa [4–7] (QuickCalcs by GraphPad Software Inc. 2002–2005). The NR was externally tested for accuracy in the use of the CTAS guidelines using a random case subset and independent review by a Master's prepared ED nurse educator (RM) with research experience in CTAS Guidelines (both adults and pediatrics) and inter-observer reliability testing.

Focus groups chose, by consensus, five Indicator Groups for triage performance review that were felt to represent common and clinically important Chief Complaints (CCs). This was done by grouping selected similar CCs from the CEDIS CC list: Chest Pain (adult chest pain cardiac features or noncardiac features from the Cardiovascular list), Headache (Neurological list item), Respiratory Illness (any item from Respiratory CC list), Musculoskeletal Injury (any item from Orthopedic or Trauma Lists), and Performance of the Pain-Scale Modifier when indicated. One data integrity indicator was also chosen and evaluated separately: Insufficient Data (information insufficient to give CTAS score by CTAS guidelines as judged by the NR, e.g., missing historical features or pertinent vital signs). Data were collected in aggregate fashion to test the inclusiveness and performance of the chosen indicators. Multiple indicators were classified as CC #1, #2, and #3. A second CC permitted either CC to include the patient in a count for the appropriate indicator. A second CC was infrequent; there were no charts with 3 CCs. For example, a CC of musculoskeletal injury, upper extremity, with a missing pain-scale would count for both pain-scale indicated but not done and insufficient data for triage, reflecting frequency counts of specific indicator problems.

## 3. Results

Kappa to establish external validity for the NR compared to the RM was 0.94, or “very good” agreement. There were 2838 patients in Trial 1 and 3091 in Trial 2 (Table 1). The distribution of CTAS scores, rate of Insufficient Data (Table 2), rate of Inconsistent Triage (Table 3), and absolute and weighted Kappa values were similar between the two trials. Weighted (absolute, CI) Kappas for Trial 1 (0.596 (0.48, 95% CI 0.45–0.52)) and for Trial 2 (0.604 (0.49, 95% CI 0.46–0.53)) revealed “moderate” to “good” strength of agreement.

Pain scale was documented in only 1019 (42.8%) and 858 (30.9%) of indicated visits (Table 4). The chosen clinical indicators captured 68% (Trial 1) and 62% (Trial 2) of all patients (Table 5). Of these, 20–65% had insufficient data for triage, with the highest group being Musculoskeletal Injury at 55% and 65%. The rate of inconsistent triage was 14–16% for those charts with sufficient data to perform reviewer CTAS, comparable between the two trial groups (Table 6). The most common reason for inconsistency in CTAS scoring was inappropriate interpretation of the pain scale; missing vital signs was also common (where this error was not sufficient to deem the case as insufficient data to determine a CTAS score by the reviewer (Table 7)). Undertriage was more common than overtriage and tended to reflect CTAS 2 and 3 being labeled 3 and 4, respectively. Overtriage tended to reflect CTAS 5 patients being scored as CTAS 4.

## 4. Discussion

Extra training of the NR to establish the role as a reference standard was successful and produced a very high level of inter-observer agreement (Kappa) with the RM, supporting the NR role as a proxy for CTAS accuracy according to national guidelines. Six key clinical indicators captured over 60% of visits, demonstrating that a small number of easily grouped indicators capture a large proportion of total visits for review. This will allow a nurse reviewer to be trained to perform retrospective chart reviews of triage at a level of complexity easily managed by a community hospital, using simple indicators and the CAEP/NENA training materials.

However, interrater reliability between the NR and the RNs was in the borderline “good” range. Large gaps in data collection were concerning: if data issues are a common occurrence in other EDs, then continuing education efforts need to be directed toward both CTAS guidelines education and compliance. CTAS education and staff turnover did not appear to influence results over time on reliability, rates of insufficient data, and inconsistent triage. Our study may reflect the level of reliability and performance accuracy expected without a persistent, structured effort to improve proficiency [9–12]. Without a more intensive audit and feedback system CTAS performance remained static. There may be a level of variability in the CTAS for which didactic training is insufficient to generate reliably good performance.

Tendencies toward over- and undertriage likely reflect the phenomenon of “regression toward the mean” [5, 6]. New teaching guidelines have recently been released [13, 14]; training within a knowledge translation process that includes an emphasis on compliance may be needed to achieve good scale performance (see http://www.caep.ca/resources).

Most studies examining CTAS performance have occurred in tertiary care, highly controlled settings, with dual triage observations. Health care facilities in Canada often do not have the funding to pursue sophisticated analysis. This study reviews actual CTAS performance in a community setting, using an easily understood, simple, and inexpensive methodology to measure reliability and a proxy for accuracy. Two major issues were identified giving the information available to the NR: (1) triage scores were inconsistent with CTAS guidelines up to 30% of the time; however (2) incomplete data and missing pain scales in a large percentage of patients were a bigger problem. ED staff must carefully maintain CTAS data quality and compliance with regular training; otherwise one may not reasonably expect CTAS to perform consistently well clinically. This would address the large number of incomplete data cases, which by definition are inaccurate risking clinical error. An analysis of triage variance is not possible in these cases, with the attendant problem of unnecessary practice variability. More capture would better define the K, improve the process of data entry, and hopefully lead to improved interobserver reliability and the standardization of care with better outcomes.

Another issue is the widespread use of Kappa statistics to examine reliability. Despite gaps in triage performance our Kappa values were surprisingly good, revealing the importance of understanding this statistic in context. Multiple measures using specific indicators may actually be superior when evaluating adherence to CTAS guidelines.

One benefit of this method is its simplified grouping of common CCs and the use of a standardized and trained reviewer and process. The same principles could be used to evaluate any triage scale with standardized clinical discriminatory characteristics, well-defined data elements, and a good training manual and approach.

Study limitations included retrospective data collection and lack of a second independent observer for the patient. However, studies using prospective design or a second triage agent could artificially improve accuracy and reliability by improving compliance with study methodology, since staff are aware of the study process. Including patients with insufficient data in the analysis of CTAS inconsistency may not accurately reflect CTAS-scale performance giving appropriate use. However, the purpose was to measure real life staff performance, and an intention to treat review given “best efforts” reflects reality and is less likely to overestimate true performance. While the study tested accuracy through compliance with the CTAS standards, it lacked a true objective measure of accuracy from an outcomes perspective [13]. The ability of the RM to serve as trainer and gold standard was not tested externally or against the newest 2008 CTAS guidelines revision. Accreditation of trainers and expansion to multiple ED settings is desirable.

## 5. Conclusion

With ED crowding pressure and with limited healthcare resource, hospitals require tools to reliably evaluate patient flow and system performance. CTAS and CEDIS implementation needs an achievable, reliable, and cost effective method for CTAS quality review so that departmental process and outcomes can be improved. We have demonstrated that (1) a small number of simple indicators may capture a large number of cases for review, making standardization simple and easy, (2) a nurse reviewer can be trained to review CTAS Guidelines performance, and (3) basic data compliance with frequent review and feedback may be as important as CTAS education courses for training efforts in a community ED. Further investigation with standardized trainers and audit/feedback loop, and correlation with outcomes is necessary in multiple ED settings.

## Figures and Tables

**Table 1:** Patient visits by Canadian Triage and Acuity Scale (CTAS) level.

CTAS visits	Trial 1 patients	Trial 2 patients	Trial 1% in level	Trial 2% In Level
CTAS 1	4	7	0.14%	0.23%
CTAS 2	149	136	5.25%	4.40%
CTAS 3	1010	1216	35.59%	39.34%
CTAS 4	1412	1514	49.75%	48.98%
CTAS 5	219	170	7.72%	5.50%
Unknown	44	48	1.55%	1.55%

Total patients	2838	3091		

**Table 2:** Insufficient data on chart as required by Canadian Triage and Acuity Scale (CTAS) guidelines (% of Table 1).

CTAS visits	Trial 1	Trial 2	Trial 1%	Trial 2%
CTAS 1	0	0	0.00%	0.00%
CTAS 2	14	23	9.40%	16.91%
CTAS 3	302	491	29.90%	40.38%
CTAS 4	805	918	57.01%	60.63%
CTAS 5	98	79	44.75%	46.47%
Unknown	44	48	100.00%	100.00%

Total having insufficient data	1263	1559	44.50%	50.44%

**Table 3:** Canadian Triage and Acuity Scale (CTAS) level inconsistency compared to nurse reviewer (NR).

CTAS visits	Trial 1	Trial 2	Trial 1 % of level	Trial 2 % of level
CTAS 1	1	1	25.00%	14.29%
CTAS 2	18	7	12.08%	5.15%
CTAS 3	251	227	24.85%	18.67%
CTAS 4	242	274	17.14%	18.10%
CTAS 5	23	7	10.50%	4.12%

Total Inconsistent with Reviewer	535	516	18.85%	16.69%

**Table 4:** Pain scale performance when indicated by chief complaint.

Pain scale done	Trial 1	Trial 2	Trial 1%	Trial 2%
Yes	1019	858	42.8%	30.9%
Indicated	2382	2774	83.9%	89.7%

**Table 5:** Frequency of the presence of chosen clinical indicator.

Clinical indicator	Trial 1	Trial 2	Trial 1%	Trial 2%
Pain	453	499	16.0%	16.1%
Chest pain	219	194	7.7%	6.3%
Musculoskeletal injury	672	777	23.7%	25.1%
Respiratory illness	527	379	18.6%	12.3%
Headache	69	60	2.4%	1.9%

Total indicators found	1940	1909	68.4%	61.8%

**Table 6:** Frequency of clinical indicator Canadian Triage and Acuity Scale (CTAS) inconsistency compared with NR (inconsistency, inconsistency/total: Table 5 as %).

Clinical indicator	Trial 1	Trial 2	Trial 1%	Trial 2%
Pain	71	51	15.7%	10.2%
Chest pain	54	38	24.7%	19.6%
Musculoskeletal pain/injury	91	71	13.5%	9.1%
Respiratory illness	78	86	14.8%	22.7%
Headache	22	18	31.9%	30.0%

Total inconsistent	316	264	16.3%	13.8%

Total indicator patients	1940	1909		

**Table 7:** Reasons for Canadian Triage and Acuity Scale (CTAS) inconsistency.

Reason	Trial 1	Trial 2	Trial 1%	Trial 2%
Pain scale higher/lower	203	156	32%	25%
No/improper peak flow	20	2	3%	0%
Over triage	104	150	17%	24%
Under triage	208	196	33%	32%
Missing vital signs	90	110	14%	18%

Total	627	614		

## References

[1] Beveridge R, Clarke B, Janes L Implementation guidelines for the Canadian ED Triage & Acuity Scale (CTAS). http://caep.ca/resources/ctas/cedis#presentingcomplaintlist.

[2] Murray M, Bullard M, Grafstein E (2004). Revisions to the Canadian Emergency Department Triage and Acuity Scale implementation guidelines. *Canadian Journal of Emergency Medicine*.

[3] Innes G, Murray M, Grafstein E (2001). A consensus-based process to define standard national data elements for a Canadian emergency department information system. *Canadian Journal of Emergency Medicine*.

[4] Manos D, Petrie DA, Beveridge RC, Walter S, Ducharme J (2001). Inter-observer agreement using the Canadian Emergency Department Triage and Acuity Scale. *Canadian Journal of Emergency Medicine*.

[5] Grafstein E (2004). Close only counts in horseshoes and … triage?. *Canadian Journal of Emergency Medicine*.

[6] Beveridge R, Ducharme J, Janes L, Beaulieu S, Walter S (1999). Reliability of the Canadian Emergency Department Triage and Acuity Scale: interrater agreement. *Annals of Emergency Medicine*.

[7] Maningas PA (2006). The Soterion Rapid Triage Systemml: evaluation of inter-rater reliability and validity. *Journal of Emergency Medicine*.

[8] Grafstein E, Bullard MJ, Warren D, Unger B (2008). Revision of the Canadian Emergency Department Information System (CEDIS) presenting complaint list version 1. *Canadian Journal of Emergency Medicine*.

[9] Göransson K (2005). Accuracy and concordance of nurses in emergency department triage. *Scandinavian Journal of Caring Sciences*.

[10] Considine J (2007). Do knowledge and experience have specific roles in triage decision-making?. *Academic Emergency Medicine*.

[11] Göransson KE (2006). Emergency department triage: is there a link between nurses’ personal characteristics and accuracy in triage decisions?. *Accident and Emergency Nursing*.

[12] Edwards B (2007). Walking in—Initial visualisation and assessment at triage. *Accident and Emergency Nursing*.

[13] Bullard MJ, Unger B, Spence J, Grafstein E (2008). Revisions to the Canadian Emergency Department Triage and Acuity Scale (CTAS) adult guidelines. *Canadian Journal of Emergency Medicine*.

[14] Warren DW, Jarvis A, LeBlanc L, Gravel J (2008). Revisions to the Canadian Triage and Acuity Scale Pediatric Guidelines (PaedCTAS). *Canadian Journal of Emergency Medicine*.

[15] Howlett MK, Wolfe H, Bartlett C, Burris D (2008). a method for review of accuracy and reliability of Canadian Emergency Department Triage Acuity Scale (CTAS) based triage process in a community emergency department setting: starting the quality improvement processs. *Annals of Emergency Medicine*.

